# Characterization of Impact Ionization Coefficient of ZnO Based on a p-Si/i-ZnO/n-AZO Avalanche Photodiode

**DOI:** 10.3390/mi11080740

**Published:** 2020-07-30

**Authors:** Gaoming Li, Xiaolong Zhao, Xiangwei Jia, Shuangqing Li, Yongning He

**Affiliations:** 1 School of Microelectronics, Faculty of Electronic and Information Engineering, Xi’an Jiaotong University, No. 28 Xianning West Road, Xi’an 710049, China; zhaoxiaolong@xjtu.edu.cn (X.Z.); jixiangwei@stu.xjtu.edu.cn (X.J.); lishuangqing@stu.xjtu.edu.cn (S.L.); 2 School of Materials Science and Engineering, Xi’an Jiaotong University, No. 28 Xianning West Road, Xi’an 710049, China

**Keywords:** p-Si/i-ZnO/n-AZO, avalanche photodiode (APD), impact ionization coefficients

## Abstract

The avalanche photodiode is a highly sensitive photon detector with wide applications in optical communication and single photon detection. ZnO is a promising wide band gap material to realize a UV avalanche photodiode (APD). However, the lack of p-type doping, the strong self-compensation effect, and the scarcity of data on the ionization coefficients restrain the development and application of ZnO APD. Furthermore, ZnO APD has been seldom reported before. In this work, we employed a p-Si/i-ZnO/n-AZO structure to successfully realize electron avalanche multiplication. Based on this structure, we investigated the band structure, field profile, Current–Voltage (I-V) characteristics, and avalanche gain. To examine the influence of the width of the i-ZnO layer on the performance, we changed the i-ZnO layer thickness to 250, 500, and 750 nm. The measured breakdown voltages agree well with the corresponding threshold electric field strengths that we calculated. The agreement between the experimental data and calculated results supports our analysis. Finally, we provide data on the impact ionization coefficients of electrons for ZnO along the (001) direction, which is of great significance in designing high-performance low excess noise ZnO APD. Our work lays a foundation to realize a high-performance ZnO-based avalanche device.

## 1. Introduction

The avalanche photodiode (APD) has a wide application in photoelectric conversion [1], especially in the areas of optical communication [2,3,4,5], imaging [6,7,8], and single photon detection [9,10,11], because it has a large avalanche gain that enables the high sensitivity [12,13]. When the detection spectral range enters the UV region, we need to utilize wide band gap materials as the absorption layer. Therefore, 4H-SiC [14,15,16,17,18,19], GaN [20,21,22,23,24,25,26,27]-based UV APDs have been demonstrated and investigated in recent years. ZnO is also an attractive wide band gap semiconductor that has a lot of advantages such as high exciton binding energy, direct band gap, low cost, ease of fabrication, and environmental friendliness [28,29,30]. Based on a simulation work, for ZnO, the ratio of the impact ionization coefficient of holes to that of electrons is rather low [31]. This has a very appealing merit that may be fully exploited to achieve high-performance, low excess noise APD devices.

Recently, some works devoted to studying the impact of ionization multiplication in ZnO/MgO [32,33,34] and TiO2/AlO_x_ [35] material systems have been reported. These works are mainly based on the metal–insulator–semiconductor–insulator–metal structure. The highest electric field occurred in the insulator layer, and an avalanche gain was observed. However, this structure has two issues in our opinion. Firstly, the high electric field is primarily in the insulator area, and the field in the semiconductor layer is relatively weak, so the separation of photo carriers and the transport of these photo carriers may have a low efficiency. Secondly, the insulator has a very large band gap; then, the threshold energy needed for impact ionization is very large. Moreover, the thickness of several tens of nanometers may be too short to accelerate the initial injected carriers. So, a traditional pin structure without these issues may be more favorable. Considering the big challenge of stable and effective p-type doping for ZnO [36,37,38], we turned to Si for a p-type material, owing to the compatibility to the highly developed CMOS technology and the availability of a high-quality Si wafer. Although the p-Si/n-ZnO heterostructure may be exhaustively investigated [39,40,41,42,43], few works have been published up to date that demonstrate the impact of ionization multiplication base on this structure. The quality of the ZnO layer and the interface between Si and ZnO are the primary constraints.

In this paper, we fabricated the p-Si/i-ZnO/n-AZO structure and realized electron avalanche multiplication in the poly-crystalline i-ZnO layer. We systematically investigated this structure with an emphasis on the avalanche multiplication process. The band structure, field profile, current voltage characteristics, threshold breakdown electric field, avalanche gain, and impact ionization coefficient were comprehensively discussed. We also investigated the influence of the width of the i-ZnO layer. The major contribution from this work is that we provide the impact ionization coefficient of electrons for ZnO through triggering an electron avalanche multiplication by visible light with a wavelength of 532 nm. Our work indicates that ZnO still holds promise to realize a high-performance APD device, and it may renew the research interest in ZnO for the application of APD.

## 2. Materials and Methods

**Figure 1:**
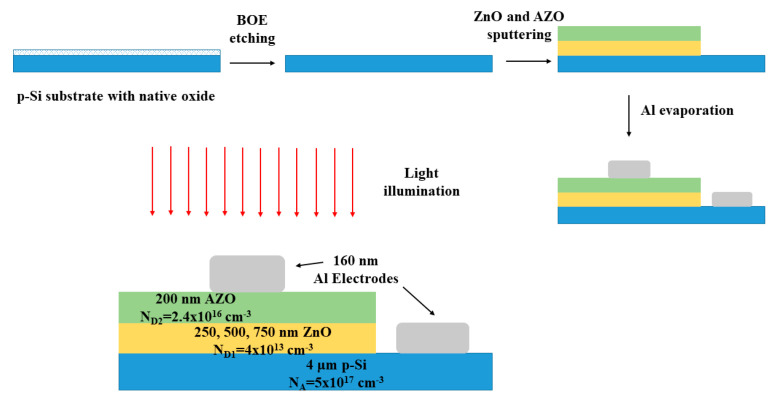
Fabrication process and structural details of the p-Si/i-ZnO/n-AZO heterostructure.

The p-Si/i-ZnO/n-AZO structure was fabricated through the process shown in Figure 1. At first, we cleansed the p+ Si wafer with acetone, ethanol, and deionized water successively; the whole cleansing process was with the help of ultrasonic oscillation. Then, we used Buffered Oxide Etch (BOE) to remove the native oxide layer on the surface of the wafer. Magnetron sputtering was exploited to deposit poly-crystalline ZnO and Al-doped ZnO (AZO) on the Si wafer in turn. The thickness and doping level of each layer are indicated in Figure 1. To investigate the dependence of performance on the width of the multiplication layer, which is the thickness of the i-ZnO layer, we tuned the thickness of the ZnO layer to 250, 500, and 750 nm, respectively. We also fabricated p-Si/n-AZO and p-Si/i-ZnO structures as references and to characterize the doping density based on a simpler pn junction structure. The aluminum electrodes were evaporated on AZO and Si, and the pattern was formed through photolithography. Since the AZO and Si are both heavily doped, Al electrodes can realize Ohmic contact with both layers.

We utilized Capacitance–Voltage (C-V) measurement to characterize the density of doping impurities for pn junctions. To eliminate the influence of interface states as much as possible, a high frequency of 1 MHz was chosen. A source measure unit was employed to do Current–Voltage (I-V) analysis for the pin structures. To probe the avalanche gain, we used a laser diode as the light source, which provides a strong illumination with the wavelength of 532 nm. We used an attenuator to reduce the light intensity, because a relatively weak light is enough to inject some charge carriers and initiate an impact ionization multiplication process.

## 3. Results and Discussion

### 3.1. Heterostructure of Si and ZnO

**Figure 2:**
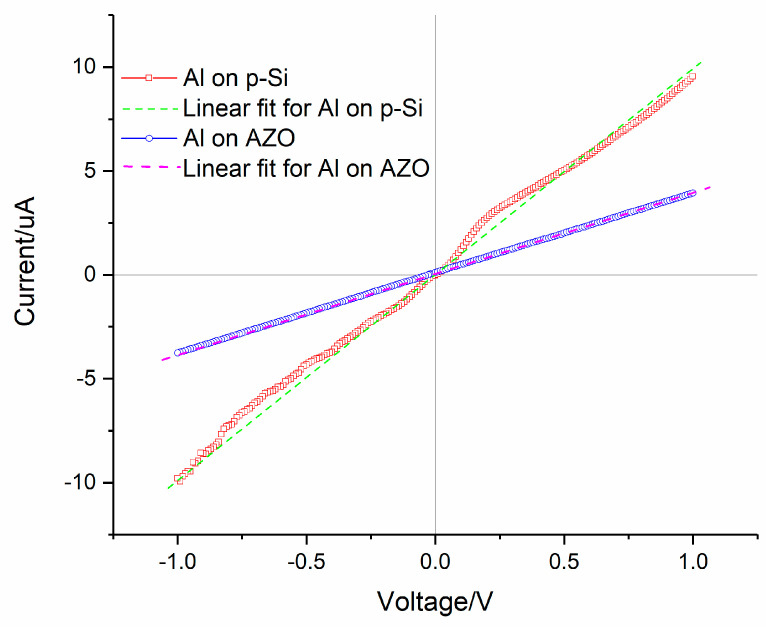
The Current–Voltage (I-V) curves for two Al electrodes on p-Si and n-AZO, respectively.

At the very beginning, we want to verify that the ohmic contacts between Al electrodes and p-Si, as well as those between Al electrodes and n-AZO, were successfully formed. We fabricated two Al electrodes with a size of 10 × 100 μm on 500 nm-thick p-Si and 500 nm-thick n-AZO films, respectively. These p-Si and n-AZO films have an identical doping concentration to those used in the p-Si/i-ZnO/n-AZO structure. The distance between electrodes is 1 cm. The I-V curves for these two setups are shown in Figure 2, from which we can see good linearity for both I-V curves. Therefore, we confirmed that ohmic contacts were formed.

As a result of lacking of durable, stable, and highly effective p-type doping for ZnO, a lot of applications for ZnO based on the pn junction need another p-type material to form a heterostructure. Silicon is a very mature and widely used semiconductor material. Its planar fabrication process, for example CMOS technology, is highly developed. Compatibility with CMOS technology and the availability of high-quality Si wafer are the benefits of choosing Si as the p-type material. Besides, the lattice mismatch between Si and ZnO is tolerable. Based on the hexagonal ZnO (a = 3.252 × 10^−10^ m) and cubic Si (a = 5.43 × 10^−10^ m) lattice parameters from the literature, we can calculate the mismatch, which is 40% in this case [28]. To alleviate the strain caused by the lattice mismatch and improve the crystalline quality of ZnO near the interface, annealing at 600 degrees Celsius for 30 min in the Ar ambient was employed after the growth of ZnO film on Si. We successfully fabricated the p-Si/i-ZnO/n-AZO structure to realize the avalanche multiplication of electrons in ZnO.

**Figure 3:**
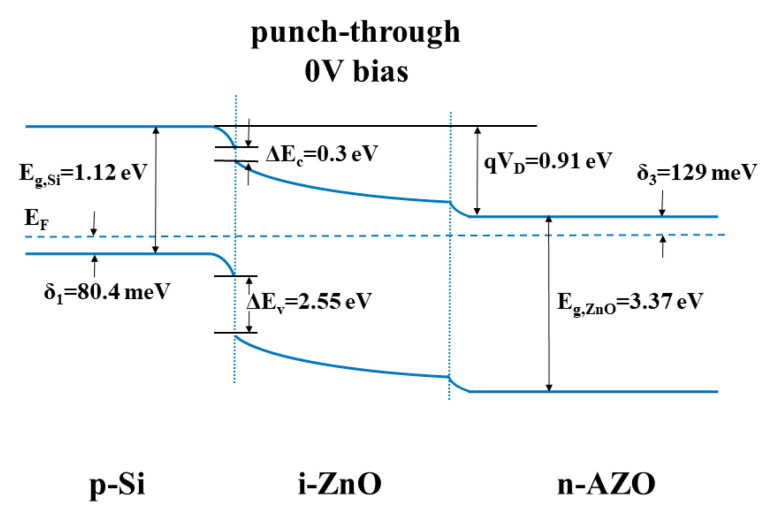
The band diagram of the p-Si/i-ZnO/n-AZO structure.

The band diagram of the p-Si/i-ZnO/n-AZO structure is shown in Figure 3. The band offset, especially for the conduction band, is crucial for analyzing the electron transport. Moreover, the bending degree of the band due to the built-in potential and applied voltage bias is also responsible for the modulation of electron transport. The position of the Fermi level in the band gap is related with the doping density, which is deduced from the C-V measurement. For p-Si substrate, the doping density N_A_ = 5 × 10^17^ cm^−3^, and it can be assumed that the doping impurities are all ionized at room temperature. Therefore, based on the following equation:

(1) $$\delta_{1}=E_{F,\mathrm{Si}}-E_{v,\mathrm{Si}}=kT\ln(N_{v,\mathrm{Si}}/N_{A})$$

where *E*_F,Si_ and *E*_v,Si_ are the Fermi level and valence band maximum (VBM) of the Si band, respectively, *k* is the Boltzmann constant, *T* is the temperature, and *N*_v,Si_ is the effective density states in the valence band of Si, which is 1.1 × 10^19^ cm^−3^ [44]. Then, we can derive the energy difference between the Fermi level and VBM in Si, which is 80.4 meV.

In a similar manner, we can deduce the energy difference between the Fermi level and conduction band minimum (CBM) in ZnO and AZO based on the following equations:

(2) $$\delta_{2}=E_{c,\mathrm{ZnO}}-E_{F,\mathrm{ZnO}}=kT\;\ln(N_{c,\mathrm{ZnO}}/N_{D1})$$

(3) $$\delta_{3}=E_{c,\mathrm{ZnO}}-E_{F,\mathrm{AZO}}=kT\;\ln(N_{c,\mathrm{ZnO}}/N_{D2})$$

where *E*_F,ZnO_ and *E*_c,ZnO_, *E*_F,AZO_ and *E*_c,AZO_ are the Fermi level and CBM for the ZnO and AZO band, respectively, *N*_c,ZnO_ is the effective density state in the conduction band of ZnO, which is 3.4 × 10^18^ cm^−3^ [44], and *N*_D1_ and *N*_D2_ are the density of ionized impurities of ZnO and AZO, respectively. The values we used here were extracted from C-V measurement, which are 4 × 10^13^ and 2.4 × 10^16^ cm^−3^ for ZnO and AZO, respectively. The energy differences in the neutral regions of ZnO (δ_2_) and AZO (δ_3_) are 295 and 129 meV, respectively. The impurity density of unintentionally doped intrinsic ZnO is determined by C-V measurement. These impurities in ZnO are native point defects such as oxygen vacancy and interstitial zinc, which serve as donors. These native defects are the origin of the self-compensation effect and the reason why p-type doping for ZnO is very challenging.

The conduction band offset ΔE_c_ is determined by the difference of the electron affinity energy between Si and ZnO, which is defined as the energy measured from the bottom of the conduction band to the vacuum level. Based on the data in the literature [44,45], the electron affinity energy values for Si and ZnO are 4.05 and 4.35 eV, respectively. Therefore,

(4) $$\Delta E_{c}=\chi_{\mathrm{ZnO}}-\chi_{\mathrm{Si}}=0.3\;\mathrm{eV}.$$

If we consider the influence of interface states, the situation could be rather complicated. Firstly, the Fermi level will be pinned by the interface states, and the bending degree of the energy band near the interface will be changed. To simplify the effect of interface, one could add a correction term to Δ*E*_c_, which is determined by the density and position in the band gap for the interface states. Here, we did not consider the influence of interface states when calculating the band offset. It is well known that the band gaps for Si and ZnO are 1.12 and 3.37 eV, respectively. We can calculate the valence band offset Δ*E*_v_ by the equation:

(5) $$\Delta E_{v}=E_{g,\mathrm{ZnO}}-E_{g,\mathrm{Si}}+(\chi_{\mathrm{ZnO}}-\chi_{\mathrm{Si}})=2.55\;\mathrm{eV}.$$

Since the doping density of Si is about 4 orders of magnitude greater than that of ZnO, the width of the depletion layer in ZnO is much greater such that the whole region of ZnO is depleted. We will discuss the width of the depletion layer in ZnO in detail afterwards. Thus, the pin diode we fabricated belongs to the punch-through type, which has a nearly constant high field in the multiplication layer. This field profile is very suitable for avalanche multiplication.

### 3.2. C-V Measurement and Field Profile

The C-V measurement is a common method to acquire the doping density and built-in potential in the semiconductor field. For the ideal pn structure, we applied a reverse bias without consideration of interface states, and the capacitance of a pn junction is mainly determined by the variation of charge in the depletion layer with the applied voltage. It is easy to deduce the equation as follows:

(6) $$\left|\frac{d1/C^{2}}{dV}\right|=\frac{2(\varepsilon_{1}N_{A}+\varepsilon_{2}N_{D})}{q\varepsilon_{1}\varepsilon_{2}N_{D}N_{A}}$$

where *N*_A_ and *N*_D_ are the doping density of the p-type and n-type layer, respectively, *ε*_1_ and *ε*_2_ are the relative permittivity of the n-type and p-type materials, respectively, and q is the electron charge. According to Equation (6), if we plot the 1/C^2^-V curve, we could derive the doping concentration from the slope.

**Figure 4:**
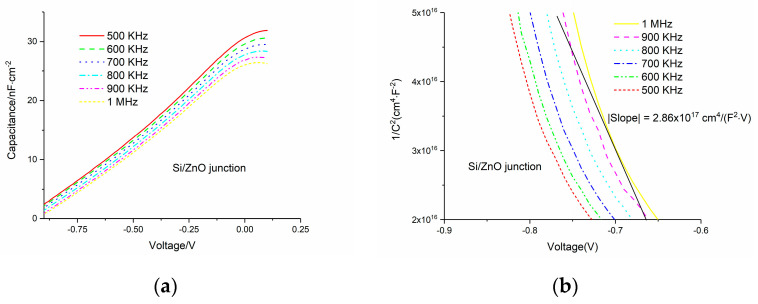
(**a**) Capacitance–voltage measurement data for the p-Si/i-ZnO junction; (**b**) 1/C^2^–V curve for p-Si/i-ZnO junction.

**Figure 5:**
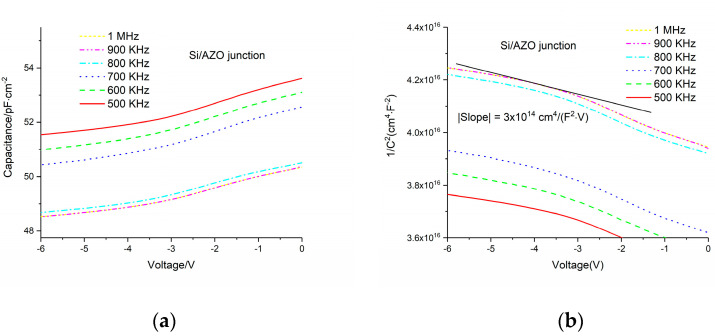
(**a**) Capacitance–voltage measurement data for p-Si/n-AZO junction; (**b**) 1/C^2^–V curve for p-Si/n-AZO junction.

**Figure 6:**
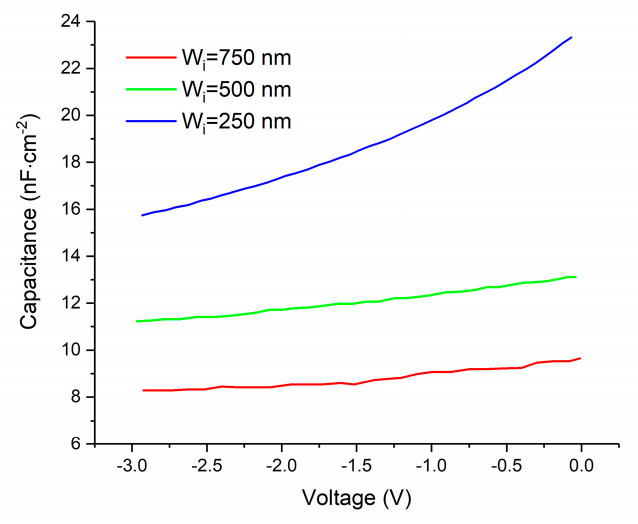
Capacitance–voltage measurement results measured with 1 MHz AC signal for p-Si/i-ZnO/n-AZO structure.

To exclude the influence of interface states, a high-frequency AC field is employed. From Figure 4a and Figure 5a, we can see that the capacitance goes down with increasing frequency, which indicates that the interface states contribute to the capacitance less at higher frequency. The reason is that the charging process through interface states is too slow to catch up with the fast varying AC field. To be more specific, the electrons and holes can be transferred between the interface states and the conduction band and valence band, respectively, depending on the position of the Fermi level. Generally speaking, the interface states that are under the Fermi level are always occupied by electrons. When the applied voltage is changing, the Fermi level will move accordingly. Then, the electric charge will flow into or out of the interface states; thus, in this case, the interfaces states behave similar to a capacitor. This charging or discharging process is usually very slow, so when the frequency of the AC voltage increases, the contribution to total capacitance from interface states gets lower. This interpretation explains the capacitance dependence on frequency well. The capacitance decreases with the increasing reverse voltage due to the increase of the width of the depletion layer. From Figure 4 and Figure 5, based on the slope of curve that we showed using a black straight line, we derived the doping densities of i-ZnO and n-AZO, which are 4 × 10^13^ and 2.4 × 10^16^ cm^−3^, respectively. As we can see either in Figure 4 or Figure 5, the slope of the curve for 1 MHz is not a single value, because the curve is not perfect straight line. Therefore, we took the average for the incline angles corresponding to the slope within the voltage range. The tangent of the average incline angle is the slope we extracted. The C-V measurement results are shown in Figure 6, from which we can deduce that the capacitance of the pin structure is mainly determined by the variation of the width of the depletion layer in AZO with the voltages.

When the doping concentration of each layer is determined, we can calculate the electric field profile based on Poisson’s equation as follows:

(7) $$\left\{ \begin{array}{l} \frac{dE}{dx}=\frac{qN_{A}}{\varepsilon_{2}},\;\mathrm{in}\;\mathrm{depletion}\;\mathrm{layer}\;\mathrm{of}\;\mathrm{Si}\\ \frac{dE}{dx}=\frac{qN_{D(1,2)}}{\varepsilon_{1}},\;\mathrm{in}\;\mathrm{depletion}\;\mathrm{layer}\;\mathrm{of}\;\mathrm{ZnO}\;\mathrm{or}\;\mathrm{AZO} \end{array}\right..$$

**Figure 7:**
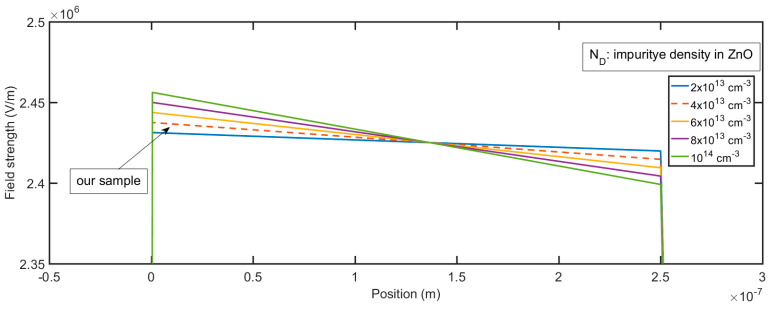
Field profile p-Si/i-ZnO/n-AZO structure for different impurity concentrations of i-ZnO layer.

The calculated electric field profile for the different impurity concentrations of the i-ZnO layer is shown in Figure 7. The relative permittivities for Si (ε_2_) and ZnO (ε_1_) that we used in the calculation are 11.9 and 9.0, respectively [44]. The variation of doping density can alter the electric field profile. Before we do the calculation, we need to verify the assumption that the i-ZnO layer is completely depleted. It can be proved by the contradiction. If the i-ZnO layer is not fully depleted, then the width of the depletion layer should be less than the thickness of the i-ZnO layer. We can calculate the width X_D_ in this case by the following equation:

(8) $$X_{D}=[\frac{2\varepsilon_{\mathrm{Si}}\varepsilon_{\mathrm{ZnO}}{\left(N_{A}+N_{D1}\right)}^{2}V_{D}}{qN_{D1}N_{A}(\varepsilon_{\mathrm{Si}}N_{A}+\varepsilon_{\mathrm{ZnO}}N_{D1})}{]}^{1/2}\approx {\left[\frac{2\varepsilon_{\mathrm{ZnO}}V_{D}}{qN_{D1}}\right]}^{1/2},\;\mathrm{For}\;N_{A}\gg N_{D1}$$

where *V*_D_ is the built-in potential for the Si/ZnO junction, which is 0.61 eV for our sample. This built-in potential was determined by the energy difference between the Fermi levels of Si and ZnO before they contact to form a heterojunction. The detailed process is outlined in the following description. From the common vacuum level, we determined the position of the CBM for Si and ZnO based on their electron affinity energy, respectively. Then, we can determine the position of the Fermi level with respect to the CBM for both of them by their impurity concentration. Finally, we calculated the difference between the Fermi levels, which is the built-in potential. Compared with the literature, this value is comparable to their results for a similar situation [46]. Based on Equation (8), we derived the width of the depletion layer, which is 1.28 μm. It is much greater than the maximum value of the i-ZnO layer, which is 750 nm. Therefore, we verified the assumption that the i-ZnO layer is fully depleted.

**Figure 8:**
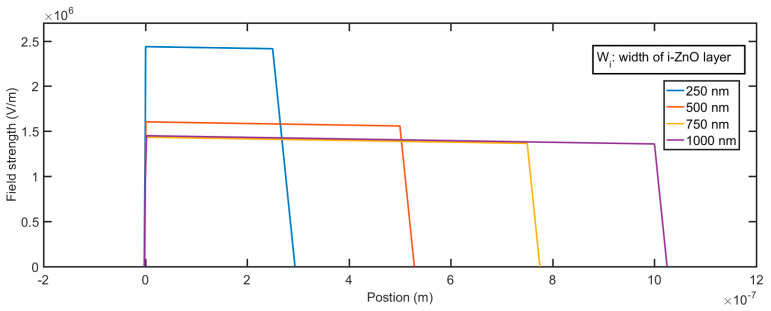
Field profile of the p-Si/i-ZnO/n-AZO structure for different widths of the i-ZnO layer.

**Figure 9:**
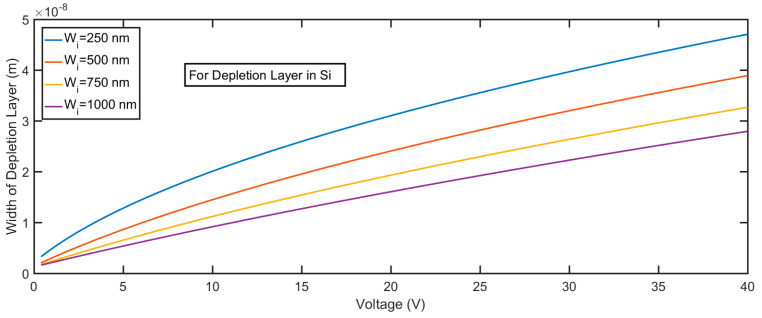
Width of the depletion layer in p-Si for the p-Si/i-ZnO/n-AZO structure under different voltages.

**Figure 10:**
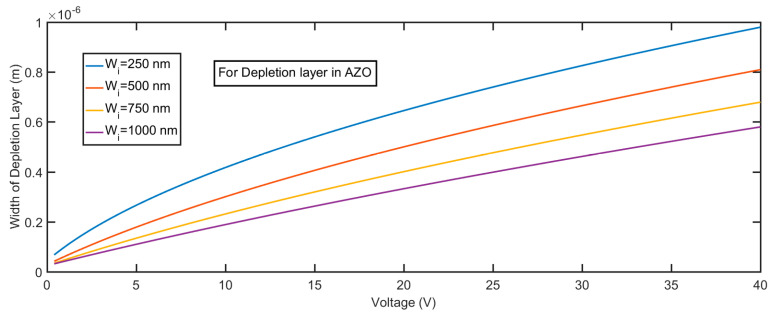
Width of the depletion layer in n-AZO for the p-Si/i-ZnO/n-AZO structure under different voltages.

The electric field profile in the depletion layer can be described by the following equations, which are the integration of Equation (7):

(9) $$\left\{ \begin{array}{l} E(x)=\frac{qN_{A}}{\varepsilon_{\mathrm{Si}}}(x+W_{p}),\;-W_{p}\leq x\leq 0\;\mathrm{in}\;p-\mathrm{Si}\\ E(x)=\frac{qN_{D1}}{\varepsilon_{\mathrm{ZnO}}}(W_{i}-x)+C_{1},\;0\leq x\leq W_{i}\;\mathrm{in}\;i-\mathrm{ZnO}\\ E(x)=C_{1}-\frac{qN_{D2}}{\varepsilon_{\mathrm{ZnO}}}(x-W_{i}),\;W_{i}\leq x\leq W_{n}\;\mathrm{in}\;n-\mathrm{AZO} \end{array}\right.$$

where *W*_p_, *W*_i_, and *W*_n_ are the width of the depletion layer for p-Si, i-ZnO, and n-AZO, respectively, and C_1_ is a constant that can be determined by the boundary conditions. The calculated field profile for different thicknesses of the i-ZnO layer is shown in Figure 8. The greatest field strength occurs at *x* = 0, which is the interface between Si and ZnO; then, the field gradually decreases with the slope, which is proportional to the impurity density of the i-ZnO layer. If the strength of the field in the multiplication layer remains very high—in other words, it decreases very slowly—it is very suitable to realize a large gain. The smaller the impurity density of i-ZnO, the more suitable the electric field profile that we can generate. Given a constant reverse bias, the greater the width of i-ZnO, the smaller the maximum field strength that we can get. Since the doping density of Si and AZO are very high, if compared with the impurity density of ZnO, the depletion layers in Si and AZO under zero voltage bias are quite narrow. From Figure 9 and Figure 10, we can see the variation of the depletion layer width in Si and AZO with reverse voltages. The width of the depletion layer in Si is in the range of several tens of nanometers, although the voltage reaches 40 V. In contrast, the width of the depletion layer in AZO increases faster with voltages; it can be several hundred nanometers, which is comparable to that in ZnO when the voltage goes high. However, the electric field strength in AZO falls much faster than that in ZnO, and the descending rate is determined by the impurity concentration. Therefore, the charge carriers transporting in these regions cannot initiate an impact ionization process, for they cannot gain enough energy from the electric field in such a short accelerating distance. It is apparent that a proper width of the multiplication layer is crucial to the ionization process, because if this value is too large, the field strength is not strong enough to accelerate the carrier to a critical velocity that can realize the impact ionization. On the contrary, if this value is too small, the accelerating distance would be too short to initiate an ionization multiplication. It is sound that we assume that the multiplication process solely happens in the i-ZnO when we simultaneously consider the electric field strength and acceleration distance.

### 3.3. Current–Voltage Characteristics and Influence of Width of i-ZnO Layer

**Figure 11:**
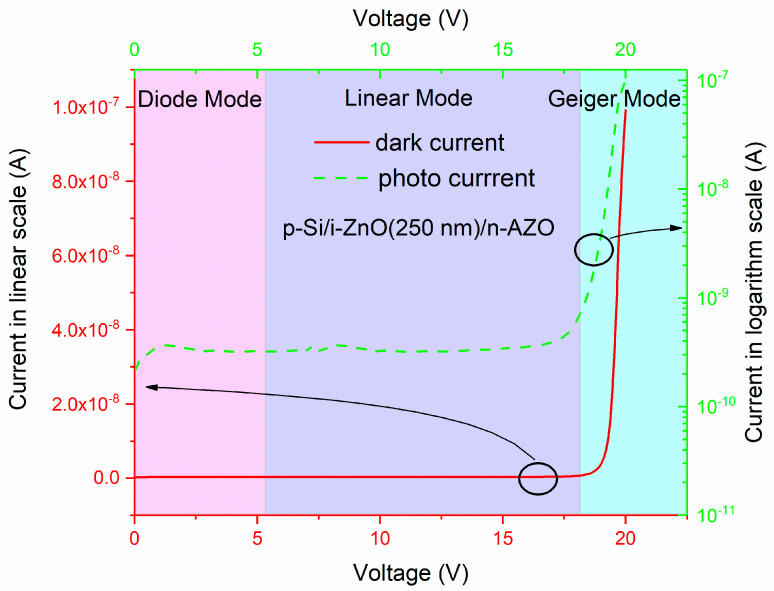
The typical I-V characteristics for our p-Si/i-ZnO/n-AZO avalanche photodiode; the green dashed line indicates the photo current, and the red solid line represents the dark current.

The strong electric field in the depletion layer is highly needed for ionization multiplication, so APD always works under reverse voltage bias. The current–voltage curve of a p-Si/i-ZnO/n-AZO structure is shown in Figure 11. The thickness for the i-ZnO layer is 250 nm. It has typical I-V characteristics for an avalanche photodiode (APD). The reverse voltage range can be broken into three regions. Within the first region, APD is in a simple diode mode without a gain or with a unit gain. In this region, the current is equal to the reverse saturation current. In the second region, APD works in a linear mode, in which the photocurrent is proportional to the light intensity, and the photocurrent decays rapidly after the light is off. The third region is related to the Geiger mode, in which an avalanche multiplication process is generated violently such that the generation rate of carriers is greater than the collection rate of the electrodes. So, we need a quenching mechanism to cease this multiplication process after the light is off. The boundary between the linear mode and Geiger mode is the critical voltage, which is called the avalanche breakdown voltage.

It is easy to deduce that the threshold energy *E*_i_ demanded for impact ionization is highly related to the band structure of the semiconductor, which serves as a multiplication layer. Regardless of whether there are two parabolic bands, three parabolic bands, or non-parabolic bands, *E*_i_ is comparable with the band gap *E*_g_. As the well-known 3/2-band-gap rule indicates, if the effective mass for electrons and holes are equal, the threshold energy *E*_i_ is equal to 3*E*_g_/2 [12]. Since the effective mass of the holes in ZnO is lacking, we took the assumption that the effective mass of the holes is equal to that of the electrons. Then, if we make a sound guess for the mean free path of the carrier, which is defined as the distance that the carrier moves between two collisions with phonons, we could roughly derive the threshold electric field strength required for impact ionization multiplication. The mean free path can be expressed as the product of the carrier velocity and the scattering time. The scattering time is the reciprocal of the scattering rate. Based on the aforementioned analysis, the threshold field strength can be described as follows:

(10) $$E_{\mathrm{BR}}=\frac{{\left(3m^{*}E_{g}\right)}^{1/2}\cdot R(3E_{g}/2)}{q}$$

where m* is the effective mass of the charge carrier, which is equal to 0.27 *m*_0_ for ZnO [44]. *m*_0_ is the electron rest mass. *q* is the electron charge. R(3*E*_g_/2) is the scattering rate for the carrier with the energy, which is equal to 3*E*_g_/2. We substitute the quantities in Equation (10) with realistic values; then, we acquire that *E*_BR_ is equal to 4.2 × 10^5^ V/cm. The R(3*E*_g_/2) we used is 1.1 × 10^13^ s^−1^ from the reference [31].

**Figure 12:**
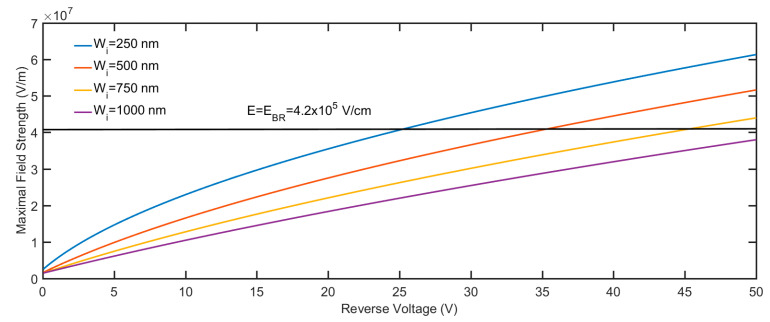
The maximum electric field strength at various reverse voltages.

We calculated the maximum electric field strength at various reverse voltages at the interface between ZnO and Si for different i-ZnO layer thicknesses. The calculated results are shown in Figure 12. As we can see, given the same reverse voltage applied, the thicker the i-ZnO layer, the smaller the maximum electric field we can get. The reverse voltages corresponding to *E*_BR_ are avalanche breakdown voltages; for the i-ZnO layer with different thicknesses, they are 25 V (250 nm), 35 V (500 nm), and 45 V (750 nm), respectively. Compared with the I-V curves for the p-Si/i-ZnO/n-AZO APD, the experimental results are smaller than the calculated results. Possible reasons for this discrepancy could be that the scattering rate we used is greater than that in the realistic situation, and the assumption and approximation may bring some errors.

### 3.4. Avalanche Gain and Ionization Coefficient

We utilized a laser diode with wavelength of 532 nm as the light source to investigate the photo response and avalanche gain of our p-Si/i-ZnO/n-AZO APD. Although the light firstly shines on the AZO side, which is on the top of the device, photocarriers will be generated in the p-Si, which is the bottom layer of the device. The reason is that the wavelength of 532 nm is much longer than the cutoff wavelength of ZnO and AZO; thus, this green light will pass through the ZnO and AZO layer without much loss. The photocarrier injection happens in the p-Si layer, and the minority carrier electrons will drift to the ZnO layer under the strong built-in electric field. So, these photogenerated electrons are the initial electrons that will be accelerated in a high-field ZnO layer and triggered the impact ionization multiplication. Based on this analysis, we can draw two important conclusions: first, the impact ionization multiplication happens in the i-ZnO layer; second, this avalanche multiplication is an electron-dominant process, and the contribution from holes can be neglected. Moreover, from the calculated results in the literature [31], we know that for ZnO, the ionization coefficient of the electrons (α) is much greater than that of the holes (β). This also supports the conclusion that we can omit the influence of the holes and solely consider the electrons’ impact ionization.

**Figure 13:**
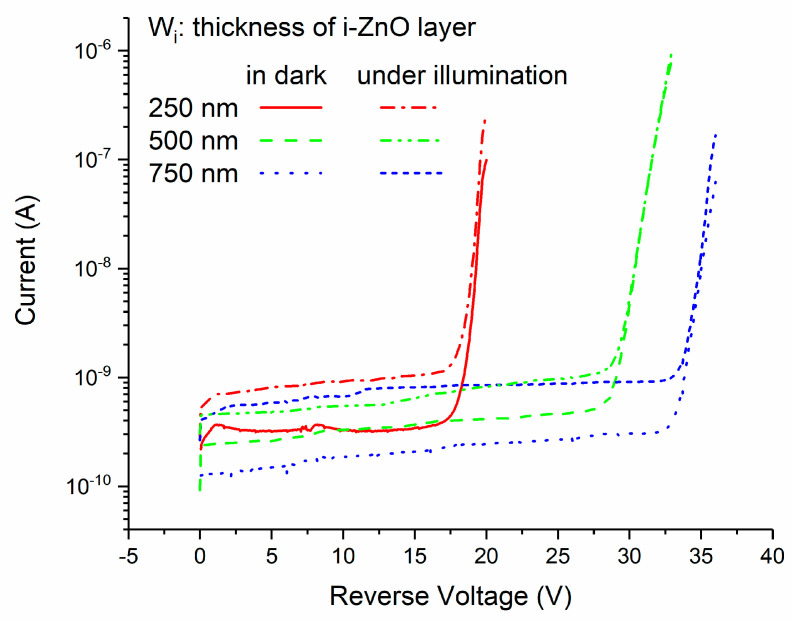
The I-V characteristics of p-Si/i-ZnO/n-AZO APD in dark and under illumination.

**Figure 14:**
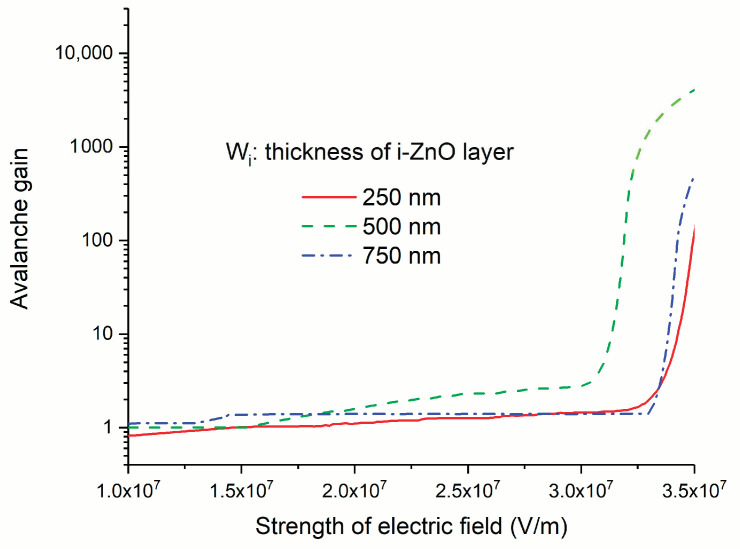
The avalanche gain at various reverse voltages.

The I-V curves of the APD under illumination are shown in Figure 13. The avalanche gain can represent the degree to which the photocurrent is magnified through impact ionization multiplication. The definition is based on the following equation:

(11) $$M=\frac{I_{\mathrm{MP}}-I_{\mathrm{MD}}}{I_{P}-I_{D}}$$

where *I*_P_ and *I*_D_ are the photocurrent and dark current without multiplication, respectively, and *I*_MP_ and *I*_MD_ are the photocurrent and dark current with avalanche multiplication. It is apparent that the ionization multiplication is highly dependent on the strength of electric field; thus, the avalanche gain is a function of reverse voltage. We calculated the avalanche gain using Equation (11). The result is shown in Figure 14. When we calculated the gain, we assumed that the gain is a unit at 5 V and used the photocurrent and dark current at 5 V for the denominator of the right part of Equation (11). From the relationship between the avalanche gain and the electric field strength, we can estimate that the breakdown voltage is about 3.3 × 10^5^ V/cm.

**Figure 15:**
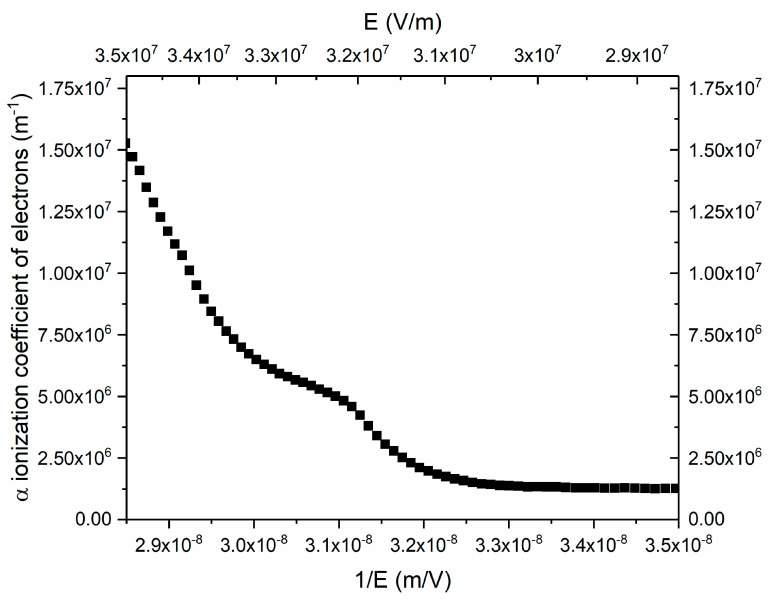
The impact ionization coefficients of electrons.

Ionization coefficients are crucial parameters that mean how many electrons and holes are created when the initial carrier travels along the electric field for a unit distance. They are related with the performance of APD such as the multiplication gain, excess noise, threshold energy, and gain–bandwidth product. When one designs a high-performance APD, the ionization coefficient is an important factor that should always be kept in mind. In a pin structure, the ionization coefficients are independent of position; therefore, the avalanche gain can be expressed using ionization coefficients as the following equation shows:

(12) $$M=\frac{\left(1-\beta /\alpha )\exp[\alpha W_{D}(1-\beta /\alpha )\right]}{1-(\beta /\alpha )\exp[\alpha W_{D}(1-\beta /\alpha )]}\approx \exp(\alpha W_{D}),\;\mathrm{if}\;\alpha \gg \beta$$

where *W*_D_ is the width of the multiplication layer. According to Equation (12), we calculated the ionization coefficient of electron (α) for APD with i-ZnO layers with different thicknesses. The calculation process is as follows. We firstly calculated the avalanche gain under various voltages; then, based on the relationship between the ionization coefficient and avalanche gain, we derived the ionization coefficient under these voltages. In an ideal situation, the avalanche gain and ionization coefficient should have been independent of the specific structural parameters of the punch-through pin structure. They are mainly determined by what material is utilized as a multiplication layer. However, owing to the nonuniformity of the ZnO layer, the avalanche gain and ionization coefficient under a given electric field extracted from the I-V curves exhibit a little discrepancy for different ZnO layer thicknesses. Therefore, we averaged the ionization coefficients derived from the pin structure with different ZnO layer thicknesses to minimize the extraction error. The results are shown in Figure 15. It should be noted that these ionization coefficients correspond to the case in which the electric field is along the (001) direction of ZnO, because our ZnO sample is highly c-axis oriented and the electric field is along the c-axis, which is the (001) direction. The results represented herein show that p-Si/i-ZnO/n-AZO is a promising structure to realize high-performance APD.

## 4. Conclusions

In this paper, we have fabricated a p-Si/i-ZnO/n-AZO structure and realized avalanche multiplication in the poly-crystalline i-ZnO layer based on this structure. The band structure of this p-Si/i-ZnO/n-AZO heterostructure was systematically investigated. Based on C-V measurement, the ionized impurity density of the unintentionally doped ZnO layer is 4 × 10^13^ cm^−3^, while the doping density of the AZO layer is 2.4 × 10^16^ cm^−3^. This i-layer with rather low impurity density is very important for a suitable field profile and ionization multiplication. We calculated the field profile for our p-Si/i-ZnO/n-AZO structure and investigated how the impurity density of the i-ZnO layer and the width of the i-ZnO layer affect the field profile. We chose three values, which are 250, 500, and 750 nm for the width of the i-ZnO layer. The calculated results show that the p-Si/i-ZnO/n-AZO structure of the parameters mentioned before is a punch-through type pin APD, which holds a very high field in the i layer. According to the field profile and width of the depletion layer in AZO and Si, an avalanche multiplication process only happened in the i-ZnO layer. We proposed an equation that roughly estimates the threshold electric field strength for the avalanche breakdown and breakdown voltage for different i-ZnO layer widths. The threshold electric field strength for avalanche breakdown for our pin structure is 3.3 × 10^5^ V/cm. To initiate the electron dominant avalanche ionization multiplication process in the i-ZnO layer and derive the ionization coefficient of electrons, we used 532 nm light from a laser diode to illuminate the pin APD. The photocurrent is greatly enhanced when the ionization multiplication is triggered at high voltages. A large avalanche gain was observed, and its variation with the electric field strength was investigated. Then, we derived the impact ionization coefficient of electrons for ZnO along the (001) direction, which is a very significant parameter for APD design and optimization. Our work provides important data for high-performance APD based on an Si/ZnO heterostructure, and it also indicates that the p-Si/i-ZnO/n-AZO structure is a promising option to realize a high-performance avalanche-type device. It may renew the research interest in the realization of APD using ZnO.

## References

[1] Campbell J.C. (2016). Recent Advances in Avalanche Photodiodes. J. Lightwave Technol..

[2] Campbell J.C., Kaminow I., Li T., Wilner A.E. (2008). Advances in photodetectors. Optical Fiber Telecomunications, Part A: Components and Subsystems.

[3] Xu Z.Y., Sadler B.M. (2008). Ultraviolet communications: Potential and state-of-the-art. IEEE Commun. Mag..

[4] Assefa S., Xia F.N.A., Vlasov Y.A. (2010). Reinventing germanium avalanche photodetector for nanophotonic on-chip optical interconnects. Nature.

[5] Kato K. (1999). Ultrawide-band/high-frequency photodetectors. IEEE Trans. Microw. Theory Tech..

[6] Bertone N., Clark W. (2007). Avalanche photodiode arrays provide versatility in ultrasensitive applications. Laser Focus World.

[7] Renker D., Lorenz E. (2009). Advances in solid state photon detectors. J. Instrum..

[8] Woodson M.E., Ren M., Maddox S.J., Chen Y.J., Bank S.R., Campbell J.C. (2016). Low-noise AlInAsSb avalanche photodiode. Appl. Phys. Lett..

[9] Hayden O., Agarwal R., Lieber C.M. (2006). Nanoscale avalanche photodiodes for highly sensitive and spatially resolved photon detection. Nat. Mater..

[10] Faramarzpour N., Deen M.J., Shirani S., Fang Q. (2008). Fully integrated single photon avalanche diode detector in standard CMOS 0.18-mu m technology. IEEE Trans. Electron. Devices.

[11] Lacaita A., Francese P.A., Zappa F., Cova S. (1994). Single-Photon Detection Beyond 1-Mu-M-Performance of Commercially Available Germanium Photodiodes. Appl. Optics.

[12] Capasso F., Tsang W.T. (1985). Physics of avalanche photodiodes. Lightwave Communications Techonolgy Part D, Photodetectors.

[13] Stillman G.E., Wolfe C.M., Willardson R.K., Beer A.C. (1977). Avalanche photodiodes. Infrared Detectors II.

[14] Su L.L., Zhou D., Lu H., Zhang R., Zheng Y.D. (2019). Recent progress of SiC UV single photon counting avalanche photodiodes. J. Semicond..

[15] Hu J., Xin X.B., Li X.Q., Zhao J.H., VanMil B.L., Lew K.K., Myers-Ward R.L., Eddy C.R., Gaskill D.K. (2008). 4H-SiC visible-blind single-photon avalanche diode for ultraviolet detection at 280 and 350 nm. IEEE Trans. Electron. Devices.

[16] Chen X.P., Zhu H.L., Cai J.F., Wu Z.Y. (2007). High-performance 4H-SiC-based ultraviolet p-i-n photodetector. J. Appl. Phys..

[17] Cai X.L., Zhou D., Yang S., Lu H., Chen D.J., Ren F.F., Zhang R., Zheng Y.D. (2016). 4H-SiC SACM Avalanche Photodiode with Low Breakdown Voltage and High UV Detection Efficiency. IEEE Photonics J..

[18] Bai X.G., Guo X.Y., Mcintosh D.C., Liu H.D., Campbell J.C. (2007). High detection sensitivity of ultraviolet 4H-SiC avalanche photodiodes. IEEE J. Quantum Electron..

[19] Zhou X.Y., Tan X., Lv Y.J., Wang Y.G., Li J., Han T.T., Guo H.Y., Liang S.X., Zhang Z.H., Feng Z.H. (2019). 8 × 8 4H-SiC Ultraviolet Avalanche Photodiode Arrays with High Uniformity. IEEE Electron. Device Lett..

[20] Reddy P., Breckenridge M.H., Guo Q., Klump A., Khachariya D., Pavlidis S., Mecouch W., Mita S., Moody B., Tweedie J. (2020). High gain, large area, and solar blind avalanche photodiodes based on Al-rich AlGaN grown on AlN substrates. Appl. Phys. Lett..

[21] Ji D., Ercan B., Benson G., Newaz A.K.M., Chowdhury S. (2020). 60 A/W high voltage GaN avalanche photodiode demonstrating robust avalanche and high gain up to 525K. Appl. Phys. Lett..

[22] Nikzad S., Hoenk M., Jewell A.D., Hennessy J.J., Carver A.G., Jones T.J., Goodsall T.M., Hamden E.T., Suvarna P., Bulmer J. (2016). Single Photon Counting UV Solar-Blind Detectors Using Silicon and III-Nitride Materials. Sensors.

[23] Verghese S., McIntosh K.A., Molnar R.J., Mahoney L.J., Aggarwal R.L., Geis M.W., Molvar K.M., Duerr E.K., Melngailis I. (2001). GaN avalanche photodiodes operating in linear-gain mode and Geiger mode. IEEE Trans. Electron. Devices.

[24] Carrano J.C., Lambert D.J.H., Eiting C.J., Collins C.J., Li T., Wang S., Yang B., Beck A.L., Dupuis R.D., Campbell J.C. (2000). GaN avalanche photodiodes. Appl. Phys. Lett..

[25] Yang B., Li T., Heng K., Collins C., Wang S., Carrano J.C., Dupuis R.D., Campbell J.C., Schurman M.J., Ferguson I.T. (2000). Low dark current GaN avalanche photodiodes. IEEE J. Quantum Electron..

[26] Zhou Q.G., McIntosh D.C., Lu Z.W., Campbell J.C., Sampath A.V., Shen H.E., Wraback M. (2011). GaN/SiC avalanche photodiodes. Appl. Phys. Lett..

[27] McClintock R., Razeghi M. (2015). Ultraviolet avalanche photodiodes. Optical Sensing, Imaging, And Photon Counting: Nanostructured Devices and Applications, Proceedings of SPIE.

[28] Ozgur U., Alivov Y.I., Liu C., Teke A., Reshchikov M.A., Dogan S., Avrutin V., Cho S.J., Morkoc H. (2005). A comprehensive review of ZnO materials and devices. J. Appl. Phys..

[29] Look D.C. (2001). Recent advances in ZnO materials and devices. Mater. Sci. Eng. B-Solid State Mater. Adv. Technol..

[30] Janotti A., Van de Walle C.G. (2009). Fundamentals of zinc oxide as a semiconductor. Rep. Prog. Phys..

[31] Bertazzi F., Penna M., Goano M., Bellotti E. (2010). Theory of high field carrier transport and impact ionization in ZnO. Conference of Oxide-based Materials and Devices, Proceedings of SPIE.

[32] Yu J., Shan C.X., Huang X.M., Zhang X.W., Wang S.P., Shen D.Z. (2013). ZnO-based ultraviolet avalanche photodetectors. J. Phys. D Appl. Phys..

[33] Yu J., Shan C.X., Qiao Q., Xie X.H., Wang S.P., Zhang Z.Z., Shen D.Z. (2012). Enhanced Responsivity of Photodetectors Realized via Impact Ionization. Sensors.

[34] Zhu H., Shan C.X., Wang L.K., Zheng J., Zhang J.Y., Yao B., Shen D.Z. (2010). Metal-Oxide-Semiconductor-Structured MgZnO Ultraviolet Photodetector with High Internal Gain. J. Phys. Chem. C.

[35] Wang W.J., Shan C.X., Zhu H., Ma F.Y., Shen D.Z., Fan X.W., Choy K.L. (2010). Metal-insulator-semiconductor-insulator-metal structured titanium dioxide ultraviolet photodetector. J. Phys. D Appl. Phys..

[36] Zhang S.B., Wei S.H., Zunger A. (2001). Intrinsic n-type versus p-type doping asymmetry and the defect physics of ZnO. Phys. Rev. B.

[37] Fan J.C., Sreekanth K.M., Xie Z., Chang S.L., Rao K.V. (2013). *p*-Type ZnO materials: Theory, growth, properties and devices. Prog. Mater. Sci..

[38] Look D.C., Claftin B. (2004). P-type doping and devices based on ZnO. Phys. Status Solidi B.

[39] Narayan J., Sharma A.K., Kvit A., Jin C., Muth J.F., Holland O.W. (2002). Novel cubic Zn_x_Mg_1−x_O epitaxial hetero structures on Si (100) substrates. Solid State Commun..

[40] Shen L., Ma Z.Q., Shen C., Li F., He B., Xu F. (2010). Studies on fabrication and characterization of a ZnO/p-Si-based solar cell. Superlattices Microstruct..

[41] Chand S., Kumar R. (2014). Electrical characterization of Ni/n-ZnO/p-Si/Al heterostructure fabricated by pulsed laser deposition technique. J. Alloy. Compd..

[42] Zhang X.M., Golberg D., Bando Y., Fukata N. (2012). n-ZnO/p-Si 3D heterojunction solar cells in Si holey arrays. Nanoscale.

[43] Li X.P., Zhang B.L., Zhu H.C., Dong X., Xia X.C., Cui Y.G., Ma Y., Du G.T. (2008). Study on the luminescence properties of n-ZnO/p-Si hetero-junction diode grown by MOCVD. J. Phys. D Appl. Phys..

[44] Sze S.M., Ng K.K. (2007). Physics of Semiconductor Devices.

[45] Sun H., Zhang Q.F., Wu J.L. (2006). Electroluminescence from ZnO nanorods with an n-ZnO/p-Si heterojunction structure. Nanotechnology.

[46] Romeo R., Lopez M.C., Leinen D., Martin F., Ramos-Barrado J.R. (2004). Electrical properties of the n-ZnO/c-Si heterojunction prepared by chemical spray pyrolysis. Mater. Sci. Eng. B.

